# Protein design under competing conditions for the availability of amino acids

**DOI:** 10.1038/s41598-020-59401-9

**Published:** 2020-02-14

**Authors:** Francesca Nerattini, Luca Tubiana, Chiara Cardelli, Valentino Bianco, Christoph Dellago, Ivan Coluzza

**Affiliations:** 1 Faculty of Physics, University of Vienna, Boltzmanngasse 5, 1090 Vienna, Austria; 2 Center for Cooperative Research in Biomaterials (CIC biomaGUNE), Basque Research and Technology Alliance (BRTA), Paseo Miramon 182, 20014 San Sebastian, Spain; 3 IKERBASQUE, Basque Foundation for Science, 48013 Bilbao, Spain

**Keywords:** Computational biophysics, Biological physics, Statistical physics

## Abstract

Isolating the properties of proteins that allow them to convert sequence into the structure is a long-lasting biophysical problem. In particular, studies focused extensively on the effect of a reduced alphabet size on the folding properties. However, the natural alphabet is a compromise between versatility and optimisation of the available resources. Here, for the first time, we include the impact of the relative availability of the amino acids to extract from the 20 letters the core necessary for protein stability. We present a computational protein design scheme that involves the competition for resources between a protein and a potential interaction partner that, additionally, gives us the chance to investigate the effect of the reduced alphabet on protein-protein interactions. We devise a scheme that automatically identifies the optimal reduced set of letters for the design of the protein, and we observe that even alphabets reduced down to 4 letters allow for single protein folding. However, it is only with 6 letters that we achieve optimal folding, thus recovering experimental observations. Additionally, we notice that the binding between the protein and a potential interaction partner could not be avoided with the investigated reduced alphabets. Therefore, we suggest that aggregation could have been a driving force in the evolution of the large protein alphabet.

## Introduction

The amino acid alphabet encoding the protein function is common to all living organisms and is the result of millions of years of evolution. It is composed of 20 letters, in contrast to the ones of other biopolymers, such as DNA and RNA, which possess 4 letters only. Such a large alphabet gives to proteins the vast variety of configurations and functions that we know so far.

The advent of computational protein evolution (also known as protein design)^1–16^ opens the possibility to address fundamental questions about the nature of the amino acid alphabet^17–20^. Protein design consists in searching for protein sequences capable of folding into a given backbone conformation. The search is usually done by point mutations while keeping the backbone structure fixed. In addition to several applications to medicine^12,14,21–23^ and material science^15,24–27^, protein design offers the possibility to explore fundamental problems of protein evolution.

One of the questions that mostly attracts the attention of the scientific community is about the universality of the 20 letters. Of course, the complex spectrum of proteins functionalities calls for a wide range of building blocks. However, could it be possible to design proteins to fold using a reduced alphabet? And, if yes, why not simply stick with such a reduced alphabet?

The early work on protein design with alphabets of different sizes was carried out for protein lattice models in which the protein chain is constrained to be on a cubic lattice. With such models it was possible to design heteropolymers with a large variety of alphabets defined by the amino acid interactions^28–37^. It became rapidly apparent that even in such simplified systems it is necessary to have a minimum number of residue types to encode the target configurations^38^. Moreover, such simple models allowed to explore the related question on how the alphabet size influences protein-protein interactions^39–42^. Finally, works done on realistic models offer substantial evidence that protein design with a minimalistic alphabet is possible^43–47^. In particular, statistical analysis of protein databases^48–54^ demonstrated that a considerable fraction of the information encoded in natural proteins could be packed into smaller efficient alphabets from 12^54^ all the way down to just 5 residue types^43,45,54–57^. However, all the mentioned studies completely neglect the possibility that a competition for the availability of amino acids may have played a role in the evolution of the protein alphabet size.

In this work, we devised a design strategy that includes such a competition to spontaneously drive the selection towards the minimal subset of residues essential for protein folding.

Our principal result is the identification of an optimal protein alphabet with the minimum number of letters, without the need of imposing neither the size nor the composition of it. The results show that for the folding of a small protein the minimum number of amino acid types needed is just 4. Incidentally, 4 is also the alphabet size of RNA that was hypothesized to be a precursor of proteins during the early stages of life. Additionally, by having a binary system, we can explore the effect of the alphabet reduction on aggregation in different protein-protein binding scenarios. From our simulations we observe that the alphabet reduction compromises the heterogeneity of the protein-protein interactions^28,36,40–42^ and binding cannot be avoided.

These results have interesting implications towards the understanding of the evolution of protein sequences and structures when the amino acid availability is taken into account. In fact, living systems are under constant pressure for using the smallest variety of amino acids as possible, e.g. to limit the resources needed to construct specialised tRNA molecules necessary for the translation process^58^. Hence, it is reasonable to assume that during the early stages of life, the protein capable of being designed with a smaller alphabet could have been advantageous. If protein aggregation was not crucial at that stage, then our results demonstrate that protein-based life could have started with an alphabet size compatible with the one of DNA and RNA. On the other hand, the simple condition of avoiding protein aggregation could be a strong driving force against alphabet reduction.

## Methods

We consider systems composed of the natural protein G structure (already successfully redesigned with several protein models^3,7^) and a competing element (a mould of a part of protein G, that mimics with a surface-like shape a potential binding site of a larger protein). Both proteins are represented with the caterpillar coarse-grain model, which has been successfully tested to design and refold natural and artificial proteins^7,9^ including the protein G.

In the following we will use the denominations: protein G referring to both natural structure and sequence as stored in the PDB with the ID 1pgb; protein $\bar{G}$ referring to an artificial sequence designed for the natural protein G structure; protein $\Gamma$ referring to the surface-like competing protein partner.

The protein $\Gamma$ is created immersing the protein G structure into a flat surface until its centre of mass (CM) reaches the desired relative height $\zeta$ with respect to it. The flat surface is pushed down creating a mould, which is kept at fixed distance $\mu =13\,{\rm{\AA }}$ from the surface of the protein G. Then, the protein G is rotated around its CM to maximise the mould surface area, which represents the binding site of a second protein. We create four moulds, each corresponding to a different value of $\zeta$ and composed by a different number of amino acids, labelled as ${C}_{{\rm{surf}}}$. The systems are characterised by $\zeta$ = (0.20, 0.40, 0.60, 0.80), thus leading to surface areas = (4717.5, 3842.2, 3051.5, 2320.5) Å^2^ and *C*_surf_ = (158, 127, 100, 78) residues respectively (see the *Modelling protein* $\Gamma$ of the Supplementary Materials SM for details). For the sake of simplicity, we call *sequence* the amino acid identities of protein $\Gamma$, although its surface-like structure is frozen and far from a polymeric chain of beads.

**Figure 1:**
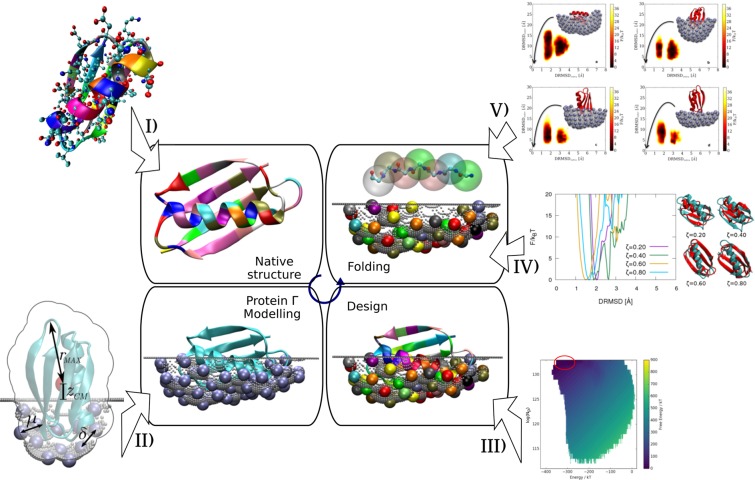
Pictorial representation of the steps employed to enforce a competition for amino acid availability between protein $\bar{G}$ and a protein $\Gamma$, and to test its effect on the folding ability of protein $\bar{G}$ in presence and absence of the artificial partner. (I) Create a Caterpillar version of the experimentally determined crystal structure of protein G (II) Shape four competing partner proteins $\Gamma$ modelled as moulds of increasing portions of the protein G. The size of the mould will influence the competition for resources, as further explained in the following sections. The larger the surface, the higher the competition. (III) Design each of the four systems considering simultaneously the proteins $\bar{G}$ and $\Gamma$. The procedure consists in searching for the ensemble of sequences that minimise the energy of both protein $\bar{G}$ and $\Gamma$ while keeping the system conformation frozen in space. The competition for the amino acids is created at this stage of our simulations. (IV) After selecting the best designed sequence (see the *Design* subsection for details about the criterion) for each system, isolate the portion relative to the protein $\bar{G}$ and test its folding ability in a single-protein folding simulation. (V) Check how the folding of the latter sequences is influenced by the presence of protein $\Gamma$ frozen in the simulation box (bearing the sequence designed concurrently to protein $\bar{G}$).

The procedure employed in the present work follows the steps pictorially represented in Fig. 1,

Once the protein $\Gamma$ modelling is complete, the structures of both proteins are frozen, with the protein G immersed into the mould $\Gamma$ and kept at distance *μ* from it (as represented in Fig. S1b). The design scheme consists of a computational exploration of the sequence space via point mutations, looking for the ones that minimise the total energy among the ones that maximise the permutations ${N}_{P}=\frac{N!}{{n}_{A}!{n}_{B}!{n}_{C}!\ldots }$ of the total amino acid composition (*N* is the total number of amino acids and $[{n}_{A},{n}_{B},{n}_{C},\ldots ]$ are the abundance on amino acids of type $A,B,C,\mathrm{.}.$ respectively). See the subsection *Design* of *Technical aspects of the methodology* in the SM for details. It is important to stress that ${N}_{P} > {N}_{P}^{\bar{G}}{N}_{P}^{\Gamma }$, where ${N}_{P}^{\bar{G}}$ and ${N}_{P}^{\Gamma }$ are the permutations of protein $\bar{G}$ and $\Gamma$ respectively. This inequality implies that, indeed, the sequences of $\bar{G}$ and $\Gamma$ are correlated, since the most heterogeneous sequence is not the one that maximises ${N}_{P}^{\bar{G}}$ and ${N}_{P}^{\Gamma }$ separately. In turns it means also that ${N}_{P}$ can be maximised without maximising ${N}_{P}^{\bar{G}}$ and ${N}_{P}^{\Gamma }$ separately, and the residues can be distributed dishomogeneously between protein and substrate.

The choice of the distance *μ* between the two proteins guarantees that, during the design, the protein-protein interaction energy is negligible. Under such conditions, the design scheme leads inherently to sequences that minimise the energy of the protein $\bar{G}$ and optimise the exposure to the solvent of each residue of protein $\Gamma$. Since protein $\bar{G}$ and $\Gamma$ are energetically uncorrelated, the coupling between the proteins is then only through the maximisation of the total permutations *N*_*P*_.

## Results

For each scenario, i.e. for each $\zeta \in (0.2,0.4,0.6,0.8)$, the design algorithm generates a basin of solutions containing approximately 10^5^ sequences. From each basin, we select the sequence with highest permutation number and lowest energy, considering it as representative of the whole basin, and use it to test the folding and binding properties. The selected protein $\bar{G}$ sequences for each scenario are shown in Table S1, while in Table S2 we show how much they differ from each other. To search for the smallest alphabet, we decided to focus on a single sequence instead of an average over a basin. Taking as a reference the centroid of the basin would have shifted the solution space towards higher energy sequences that tend to have larger alphabets.

We observe that the residues of protein $\bar{G}$ tend to adopt a limited set of letters. Moreover, increasing the protein $\Gamma$ area (and hence the number of amino acids belonging to it) reduces de facto the amino acids accessible by the protein $\bar{G}$ to minimise its energy. Hence, the fractionation of the alphabet is not caused by specific interactions between the residues but by the coupling through the maximisation of the total permutations *N*_*P*_.

We can control the competition pressure by changing the size of protein $\Gamma$. This competition leads to an effective reduced alphabet used by the protein $\bar{G}$. We observe that the effective alphabet grows from 4 to 6 letters going from larger (*ζ* = 0.20 and 0.40) to smaller $\Gamma$ proteins (*ζ* = 0.60 and 0.80) respectively. It is interesting to notice that the alphabets are made of amino acids with an average attractive pair-interaction energy and high variability in terms of the residue-solvent interactions (see Table S1 in ref. ^9^). Moreover, the alphabets differ from each other (letters *GKVY* and *GKRV* corresponding to $\zeta =(0.20,0.40)$ and *FGHKRY* common to both $\zeta =(0.60,0.80)$), and for each scenario the protein amino acids are not present in the corresponding protein $\Gamma$ sequence (see SM Fig. S11). Therefore, part of the 20 letters are segregated on the protein $\Gamma$ sequence.

Our finding shows that the design process indeed mimics a process under competition for available amino acids. It is important to stress that such competition is the results of the coupling alone as we impose neither the size nor the composition of the reduced alphabet. Hence, the particular letters that the design process chooses for protein $\bar{G}$ are presumably optimal to stabilise the folded structure. This feature is the crucial element of our design scheme that allows us to isolate the critical set of residues in our alphabet for design and folding.

Finally, we test the folding and binding properties of the designed sequences. Hence, we perform Monte Carlo simulations keeping fixed the amino acid sequence generated for each scenario, and extensively exploring the conformational space of the protein $\bar{G}$.

**Figure 2:**
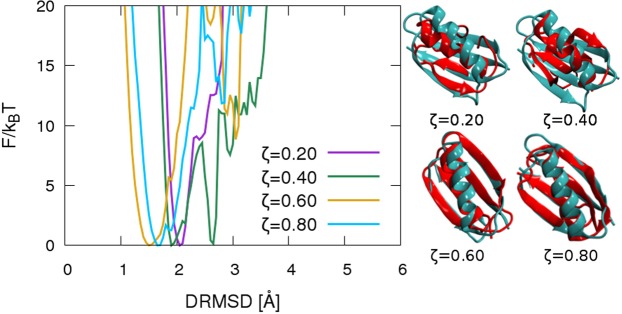
Folding free energy profiles *F*/*k*_*B*_*T* of single protein (only protein $\bar{G}$, no protein $\Gamma$) at reduced temperature 0.55 as a function of DRMSD from the native target structure (protein G structure, PDB ID: 1pgb). Different colours correspond to protein $\bar{G}$ sequences obtained via the design procedure in the presence of the protein $\Gamma$ characterised by the $\zeta$ value specified in the key. Right hand side: configurations corresponding to the free energy minimum for each system are represented in red, compared to the native protein G (in green). $DRMSD=2.1\,{\rm{\AA }}$ for $\zeta =0.20$; $DRMSD=1.9\,{\rm{\AA }}$ for $\zeta =0.40$; $DRMSD=1.3\,{\rm{\AA }}$ for $\zeta =0.60$ and $DRMSD=1.5\,{\rm{\AA }}$ $\zeta =0.80$.

To test the selected sequences, we first examine the folding stability of the protein $\bar{G}$ alone, therefore performing a folding simulation in an empty box starting from a fully stretched configuration. Figure 2 shows the free energy profiles as a function of the distance root mean square displacement *DRMSD* (defined in Eq. S10 of SM). From previous works^7,9^, the criterion for assessing a stable fold is to observe a funnel shape of the free energy profile and a global free energy minimum for $DRMSD\le 2\,{\rm{\AA }}$. Using this criterion, we can say that all protein sequences fold back into the target configuration, although with different precision. Sequences with a larger effective alphabet fold with higher precision, as can be seen from the *DRMSD* value of the configurations corresponding to the global free energy minimum for each system (The DRMSD values correspond to 4.9; 5.5; 2.4 and 2.7 Å in *RMSD* respectively). The sequence optimised at $\zeta =0.40$ shows a secondary minimum in the free energy, corresponding to misfolded compact structures, therefore being the system less stable for the folding in the bulk. A possible explanation of such a behaviour is that the effective 4 letters protein $\bar{G}$ alphabet for $\zeta =0.40$ involves only hydrophilic residues (*GKRY*), thus leading to a lower stability.

From the described scenario, we can draw two important conclusions: firstly, design with a limited alphabet of 4 letters can produce a funnel-like folding free energy landscape; secondly, with 6 letters we recover the folding precision of previous caterpillar designs made with 20 letters^9^. Our results are consistent with the experimental observation that 6 letters are a minimal set necessary to maintain protein structure and function^43,45,54–57^.

The Random Energy Model^59–61^ provides a criterion for a heteropolymer to be designable: it has to satisfy the relation $q > \exp (\omega )$, where q is the alphabet size and $\omega$ the conformational entropy per residue. Hence, a 4 letters alphabet gives an upper bound to the conformational entropy $\omega$ of the caterpillar backbone and therefore of the more restricted natural protein backbones. Such a result is compatible with the recent observations of Cardelli *et al*.^62^ who mapped the designability phase space for a general heteropolymer decorated with directional interactions similar to the hydrogen bonds present along the protein backbone. For polymers with two directional interactions per particle the minimum alphabet measured was four, as the one presented here.

**Figure 3:**
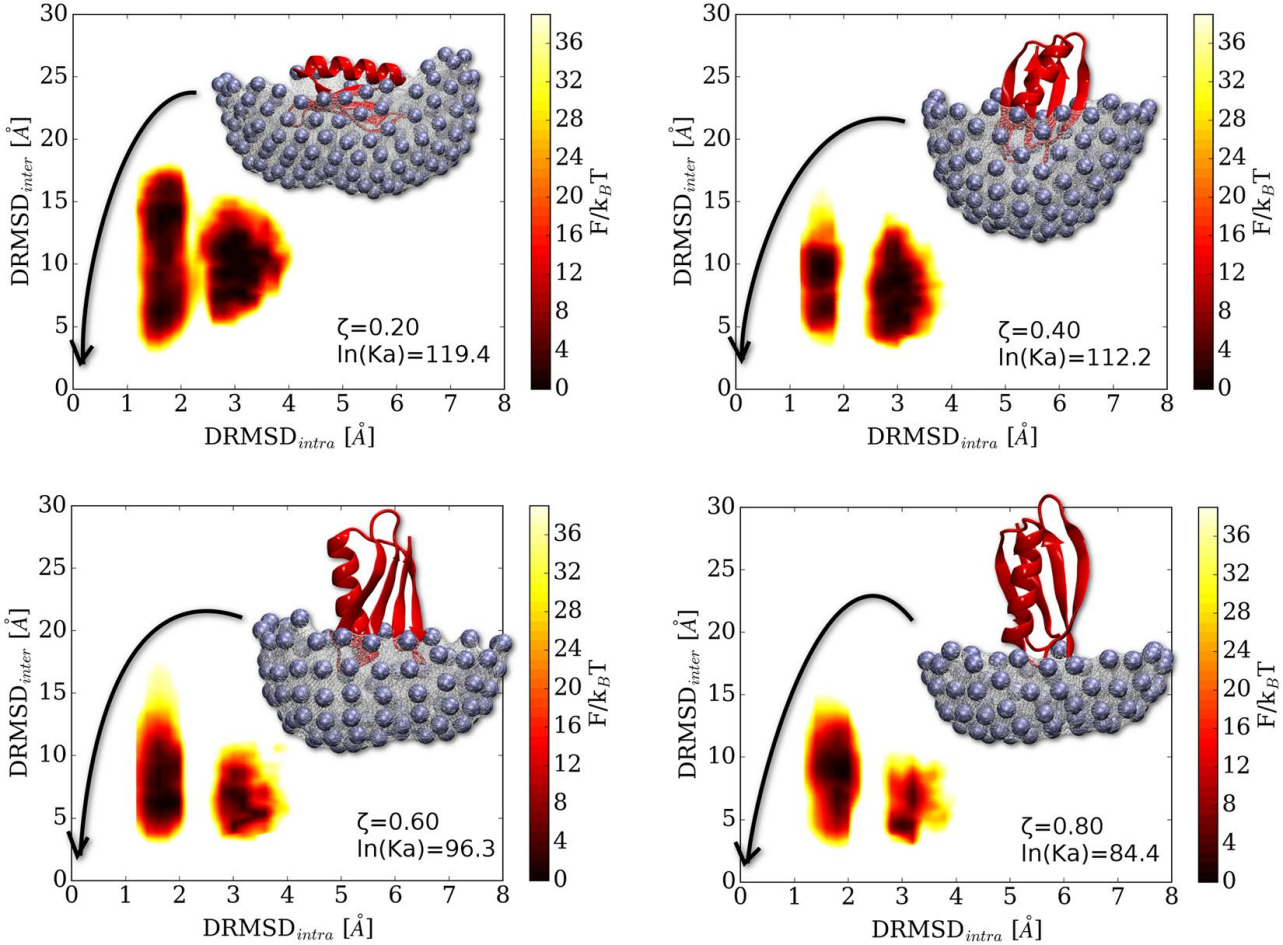
Folding free energy landscapes *F*/*k*_*B*_*T* at reduced temperature 0.76 as a function of the $DRMS{D}_{{intra}}$ distance from the native protein G as target and the $DRMS{D}_{{inter}}$ inter-protein distance from the folded protein bound to protein $\Gamma$ (configurations depicted in the panels). The binding affinity decreases along with the protein $\Gamma$ surface size, as shown by the value of the association constants *K*_*a*_ in the plot key.

To test the effect of the alphabet reduction on protein-protein interaction, we also perform folding simulations in the presence of the protein $\Gamma$, that represent a potential binding site. In Fig. 3 we plot the free energy landscape as a function of $DRMS{D}_{{intra}}$ and $DRMS{D}_{{inter}}$. $DRMS{D}_{{intra}}$ is the *DRMSD* intra protein $\bar{G}$, and uses the native protein G structure as target configuration. $DRMS{D}_{{inter}}$ is the *DRMSD* between protein $\bar{G}$ and protein $\Gamma$, and uses the folded bound configuration (shown in the insets of Fig. 3 for each scenario) as a target. This choice allows us to monitor the folding and binding properties of the system independently. Conformations that are folded and bound can be found in the bottom left corner, while folded unbound ones in the top left corner.

Additionally, we also separately check the free energy profiles as a function of $DRMS{D}_{{intra}}$ for conformations with protein $\bar{G}$ in contact with protein $\Gamma$ (see Fig. S9 in the SM) and in the bulk solution (i.e. where no inter-protein contacts are possible, see Fig. S10) in the SM. For a sketch of the definition of contact and bulk solution configurations see Fig. S6 in the SM. To verify the consistency of the two different folding simulations, we checked that the free energy profiles of configurations in the latter region correctly fold into the target structure (Fig. S10), reproducing the behaviour observed in the isolated protein folding simulations (Fig. 2).

For all scenarios, upon binding to protein $\Gamma$, we observe a significant enhancement of misfolded configurations with respect to what observed in the bulk solution (compare Figs. S9(a) and S10 in the SM). In particular, there is a considerable shift in the equilibrium towards states at $DRMSD\sim 3\,{\rm{\AA }}$ that have a free energy that is now comparable to the one of properly folded configurations. It should be noticed that natural binding sites expose much smaller surface areas then the one modelled with protein $\Gamma$. Hence, the latter effect might be mitigated considering smaller surfaces for protein $\Gamma$.

Analysing the behaviour of the binding process as a function of temperature we find that the random binding is overall very strong and it decreases while increasing the temperature. The van’t Hoff plot^63,64^ shows positive binding affinities and an exothermic process above the folding temperature (Fig. S7 SM; see Fig. S6 SM for details about the evaluation of the association constant and Fig. S8 SM for the folding temperature evaluation). At the same time, while increasing the temperature, the equilibrium shifts from partially-misfolded to fully-misfolded, indicating that the unfolding process takes place at the surface while the protein remains bound (see Fig. S9(b)). This is particularly evident for extended protein $\Gamma$ surfaces, i.e. systems characterised by $\zeta =(0.20,0.40)$. Hence, we observe a strong tendency of the protein $\bar{G}$ designed with 4 letters to absorb and aggregate on protein $\Gamma$.

Overall, this binding behaviour is an unexpected result. In the crowded cellular ambient, natural protein designed by evolution with the 20 letters alphabet are not aggregating. As such, in the present work, protein $\bar{G}$ and $\Gamma$ should not aggregate, since they interact through the total caterpillar alphabet of 18 letters. However, our design scheme imposes a segregation of few letters on the protein $\bar{G}$ sequence. We identify the following observation as a possible cause. The 4 letters alphabets (*GKVY* and *GKRY* corresponding to $\zeta =(0.20,0.40)$) have an average intra-protein residue interaction of −0.2*k*_*B*_*T*, while the average interaction of the single protein $\bar{G}$ letters with all the others, i.e. the inter-protein interaction, is much lower −0.3*k*_*B*_*T*. This makes impossible for the protein $\bar{G}$ to stabilise the folded state in contact with protein $\Gamma$. Conversely, the 6 letter alphabet (*FGHKRY* common to both $\zeta =(0.60,0.80)$) has an average intra-protein residue interaction of −0.4*k*_*B*_*T*, that is lower than the inter-protein one of −0.3*k*_*B*_*T*. This helps in stabilizing the folded structure upon binding. If, on the other end, the residues would have been properly mixed, there would be no difference between inter and intra averages, and the random interactions should be washed out by thermal fluctuations^28^. Hence, there is a fundamental pressure to increase the alphabet size and fully use it to achieve folding and avoid strong absorption.

This is an essential factor that could explain why natural proteins tend to have and use a larger alphabet than 6 letters. However, the origin of the 20 letters is still only matter of speculation. In fact many molecular process require additional chemical modification of the proteins like glycolisation that effectively increases the available pools of potential letters. Hence, it is not even accurate to consider 20 as the upper limit, that is why in this study we focused on the lower limit that has more clear definition.

In conclusion, the design procedure employed in our work has a significant segregation effect on the alphabet letters used in the protein $\bar{G}$ sequence. The larger the number of residues on the competing protein $\Gamma$, the smaller is the effective alphabet available for the protein $\bar{G}$ sequence. On the one side, the design is capable of selecting a subset of letters that still allows the folding of the protein in the bulk solution even for the smallest effective alphabet (4 letters). The precision of the folding increases with the effective alphabet size. Interestingly, the experimentally determined minimum alphabet size of 6 letters is also what we identify as minimum alphabet that recovers the design accuracy commonly obtained with a 20 letter alphabet. This implies that functionality will push the alphabet to grow. This trend could explain why reduced alphabets obtained form the analysis of natural proteins then to be larger^54^.

It is important to stress that the reduced alphabet presented here might not be the only possible solution. It would be interesting to perform a larger study of the folding sequences and generate a spectrum of possible 4 letters alphabets, and with models that include amino acids charges more explicitly.

Our results have far-reaching implications both in the field of protein design and for the understanding of protein evolution. In protein design, the possibility of using a reduced alphabet would considerably accelerate the search of the sequence space for good folders. In the field of protein evolution instead, the understanding of the smallest alphabet necessary for accurate protein design is still an open question. To the best of our knowledge, this study represents the first successful design of a full natural protein structure with a reduced alphabet of just 4 letters. Moreover, such a result offers an interesting parallelism with the 4 letter alphabet of RNA which studies speculates had a role in the early stages of life before the advent of proteins.

## Supplementary information

Supplementary information.
