# Supplementary material for: Protein design under competing conditions for the availability of amino acids
Source: Sci Rep. 2020 Feb 14;10:2684. doi: 10.1038/s41598-020-59401-9 (PMC7021711; doi:10.1038/s41598-020-59401-9)
Supplement: Supplementary file 1 — Supplementary information. [file 41598_2020_59401_MOESM1_ESM.pdf]

Supporting information for Protein design under competing conditions for availability of amino acids

Francesca Nerattini, Luca Tubiana, Chiara Cardelli, Valentino Bianco, and Christoph Dellago
Faculty of Physics, University of Vienna, Boltzmanngasse 5, 1090 Vienna, Austria

Ivan Coluzza
*CIC biomaGUNE, Paseo Miramon 182, 20014 San Sebastian, Spain**

(Dated: November 15, 2019)

TECHNICAL ASPECTS OF THE METHODOLOGY

In what follows we will describe the procedure used to simulate the competition for amino acid types between two proteins, one of which is protein G. We make use of the *de novo* design and folding techniques as described in Ref.s [1–3]. Protein design consists in the sampling of the sequence space, to the aim of predicting the ensemble of sequences that will fold in a specific target structure. Protein folding, instead, is the process by which a specific protein sequence assumes a well defined 3D structure. We tackle the problem computationally, via Monte Carlo simulations. The proteins are represented with a coarse-grained model, namely the caterpillar [1, 2], that is capable of refolding the native sequence of several proteins including protein G. The competition is implemented including in the design a protein partner, that is a surface representing an exposed region of a larger protein, not fully considered to reduce the computational cost of the simulations. To give to the surface a protein-like shape, we moulded it around the protein G.

Our choice of the procedure is motivated by the intent of investigating the competition for resources, under which the system can spontaneously select an optimal subset of amino acids, i.e. reduce the alphabet size, for the design of protein G's structure. The design scheme explained in the following subsection searches for sequences that minimise the energy of the protein and maximise the heterogeneity of the sequence relative to the surface-like partner. Indeed, this results in a high variability for the potential partner and a reduced alphabet for protein $\overline{G}$. To the solely aim of investigating the effect of a reduced alphabet on folding properties, one could have *a priori* imposed a specific subset of amino acids to be used in the single-protein design. However, our method goes beyond such an approach, since gives the opportunity of letting the system spontaneously select both the optimal alphabet size and the best subset of amino acids among all possible combinations. On the other hand, one could have achieved the same goal without the need of an explicit partner, letting the protein compete with a reservoir as in a grand canonical Monte Carlo simulation. However, we decided to include such a partner, to have the additional benefit of further investigate the influence of it in the folding ability of artificial protein $\overline{G}$.

Protein model

The caterpillar protein model reduces the amino acid structure and represent it by the backbone atoms: C, O, C_α, N, H (Fig. S1(a)). The intramolecular energy for a protein of length N has the form:

$$\begin{aligned} E_{intra-P} =& \sum_{i=1}^{N} \sum_{j>(i+2)}^{N} E_{hb}(\theta_1, \theta_2, r_{O_i H_j}) + E_{hb}(\theta_1, \theta_2, r_{O_j H_i}) + \\ &+ \sum_{i=1}^{N} \sum_{j>(i+2)}^{N} \alpha E_{sc}(r_{C\alpha_i C\alpha_j}) + \sum_{i=1}^{N} \alpha E_{HOH} E_{sol}(\Omega - \Omega_i) + \sum_{i=1}^{N} E_{bond}(r_{C_i N_i}) \end{aligned} \tag{S1}$$

where $\alpha = 1/4$ and $E_{HOH} = 0.75$ are added to balance the relative weight of different energy terms. $E_{hb}(\theta_1, \theta_2, r_{OH})$ is a $10 - 12$ Lennard-Jones potential commonly used to represent hydrogen bonds [4]:

$$E_{hb}(\theta_1, \theta_2, r_{OH}) = -\epsilon_H (\cos(\theta_1)\cos(\theta_2))^\nu \left[5 \left(\frac{\sigma}{r_{OH}} \right)^{12} - 6 \left(\frac{\sigma}{r_{OH}} \right)^{10} \right], \tag{S2}$$

being r_{OH} the distance between the hydrogen atom of the amide group and the oxygen atom of the carboxyl group of the main chain; θ_1, θ_2 the angles between the atoms COH and OHN respectively (Fig. S1(a)), and account for the hydrogen bonds directionality; $\nu = 2$; $\sigma = 2$ Å and $\epsilon_H = 3.1 K_B T$.

$E_{sc}(r_{C\alpha_i C\alpha_j})$ mimics the side chain-side chain interaction via a smoothed square-well-like isotropic potential:

$$E_{sc}(r_{C\alpha_i C\alpha_j}) = \begin{cases} \epsilon_{C\alpha_i C\alpha_j} \left[1 - \frac{1}{1+\exp^{2.5(r_h - r_{C\alpha_i C\alpha_j})}} \right] & r_{C\alpha_i C\alpha_j} > d_{HC} \\ \infty \quad r_{C\alpha_i C\alpha_j} \leq d_{HC} \end{cases}, \tag{S3}$$

where $r_{C\alpha_i C\alpha_j}$ is the distance between the $C\alpha$ atoms; $d_{HC} = 4$ Å is the hard core diameter; $r_h = 12$ Å. Eq. S3 is a sigmoid function with a flex at $r_h = r_{C\alpha_i C\alpha_j}$, where $E_{sc}(r_{C\alpha_i C\alpha_j}) = \epsilon_{C\alpha_i C\alpha_j}/2$. The terms $\epsilon_{C\alpha_i C\alpha_j}$ are the residue-residue interaction parameters of the interaction matrix and their value is taken from Tab. S1 of Ref. [1].

$E_{sol}(\Omega - \Omega_i)$ is an implicit solvent energy term that acts as an energy penalty if a hydrophobic (hydrophilic) amino acid is exposed (buried), and has the form:

$$E_{sol}(\Omega - \Omega_i) = \begin{cases} \epsilon^i_{sol}[\Omega - \Omega_i] & \Omega_i \leq \Omega \\ 0 & \Omega_i > \Omega \end{cases} \quad , \Omega_i = \sum_{j=1}^{N} E_{sc}(r_{C\alpha_i C\alpha_j}), \tag{S4}$$

where $\Omega = 21$ is a threshold for the number of contacts in the native structure above which the amino acid is considered to be fully buried, and ϵ^i_{sol} is the Dolittle hydrophobicity index [5].

$E_{bond}(r_{CN}) = k(r_{CN} - r_{CN_{ref}})^2$ is a harmonic bonding term with elastic constant $k = 20$ $K_B T \text{Å}^{-2}$, that keeps fixed the distance r_{CN}, along with the $C\hat{C_\alpha}N$ backbone angle.
For more details about the model see Ref. [1, 2]. For the protein Γ amino acids, instead, we use a single atom representation and make use of the E_{sol} and E_{sc} terms only.

Modelling protein Γ

The protein Γ is modelled as a fixed layer of amino acids, idealizing a small pocket on the surface of a much larger globular protein. The artificial protein Γ is a mould obtained by pushing the folded protein conformation on a planar mesh of self avoiding beads (self avoiding radius = 2 Å), as illustrated in Fig. S1(b). The high density of beads in the mesh prevents the protein from passing through it.

To obtain the mould, we initially generate a dense mesh in the $z = 0$ plane by placing the beads on a 2D square lattice with step 0.5 Å.

We then identify the centre of mass of the protein and use it to measure the height of the protein with respect to the plane, which is one of our main control parameters. Specifically, we consider the CM height z_{CM} normalised over the maximum distance,r_{MAX}, of a C_α from the CM of the protein, so that $\zeta = \frac{z_{CM}}{r_{MAX}}$ ranges between 0 (when the CM lies on the $z = 0$ plane) and 1.

We consider several values of ζ and for each of them we identify a protein orientation which maximise the contact surface with protein Γ according to the following procedure. We begin by aligning the minor inertia axis of the protein with the z axis and then perform a discrete set of rotations around the x and y axes. For each of these rotations, we recompute the mould, map it to a triangular mesh, and calculate the surface of the latter. The area of the surface is obtained by summing the area of each triangle formed by all the triplets of the mesh points at $z \neq 0$.

The shape of protein Γ is obtained by setting a minimum protein-mesh bead distance $\mu = 13$ Å and using it to inflate the radius of the protein C_αs, thus generating a mould by pushing downwards all mesh beads within the inflated radius. To avoid large gaps within protein Γ, we perform an iterative smoothing procedure. For each bead of the surface we compute its distance to all its neighbouring beads on the square lattice, and if this is larger than the self-avoiding radius, we shift the vertical position of the bead so to fill the gap. The value $\mu = 13$ Å ensures that protein Γ and protein G are energetically decoupled during the design process.

In order to define the sequence of protein Γ, a homogeneously distributed set of mesh points are "activated", i.e. switched from pure self avoiding beads to $C\alpha$ atoms. In order to do so, we firstly switch all the beads of the mesh to C_α, then we set a minimum surface $C_\alpha - C_\alpha$ distance $\delta = 5$ Å and loop over all bead points starting from a corner of the mesh. For each activated atom in the loop we deactivate all points within a sphere of radius δ. Clearly, after the first atom, the loop will incur both in activated and de-activated beads. The latter are simply skipped by the procedure. The value of δ is derived from the typical nearest distance between two residues in natural proteins, as explained in Fig. S2. Finally, we deactivate all the beads at $z = 0$, so that only the pocket contains C_α beads.

Since the amino acids in the $C_{\texttt{Surf}}$ set are represented by C_α atoms, the inter-protein interaction energy includes the caterpillar E_{sc} and E_{sol} terms only (see Eqs. S3 and S4). Given that protein Γ representation is limited to the surface residues, such an approximation affects the correct evaluation of the protein Γ solvent exposure term E_{sol}. Since in turns E_{sol} will influence the protein binding properties, we add an offset to the number of contacts for each amino acid of protein Γ (~ 6), to correctly account for the solvent exposure term E_{sol}. The offset was calculated in such a way that the maximal amino acid exposure is compatible to the one of natural proteins (e.g. the protein G itself described in Fig. S5). Finally, since protein Γ's conformation is fixed, we neglect all the interactions between all the pairs of the C_{surf} set.

Design

In order to enforce a competition for resources, we apply the previously mentioned design scheme. During the design, the configuration of the proteins are frozen in the geometry

defined during the modelling procedure of the protein Γ. The sequences of protein $\overline{G}$ and Γ are optimized simultaneously with mutation moves affecting all residues of the global system. The design consists in the optimisation of the residue-residue interactions for the target configuration, thus leading to the need of extensively investigate the sequence space. To this aim, we perform Virtual Move Parallel Tempering (VMPT) with adaptive umbrella sampling [3] Monte Carlo simulations, with swap and single point amino acid mutation moves along the sequence, a procedure that has been already successfully employed in protein design [1, 2, 6]. We simulate the same system at different temperatures, and swap sequences between the replicas on the fly (acceptance probability in Eq. S9), thus enhancing the overcoming of energy barriers and, therefore, improving the sampling. Moreover, at each temperature, we collect statistics using the information coming from all other replicas, according to the virtual move scheme described in Ref. [3]. In our implementation we simulate 16 replicas with a set of temperatures (10.000; 5.000; 2.000; 1.000; 0.500; 0.333; 0.250; 0.200; 0.167; 0.143; 0.125; 0.111; 0.100; 0.091; 0.083; 0.077) in units of k_B.

The best candidate sequences for the folding are the ones which minimise the total energy of the target structure among the ones maximising the total number of permutations N_p, given by:

$$N_p = \frac{N!}{\prod_{i=1}^{q} n_i!}. \tag{S5}$$

In Eq. S5 $q = 18$ is the alphabet size (proline and cysteine are not included in the design, due to their peculiar role in protein structure, which is beyond the scope of our model); N is the total number of monomers in both proteins; n_i is the total number of monomers of type i.

It is important to stress that the Hamiltonian used for the design is a simplification of Eq. S1, since none of the Monte Carlo moves involve a change in the geometry of the target conformation. Since we are not interested in the folding properties of the protein Γ itself, we neglect the intra-protein Γ interactions. It reduces to:

$$\begin{aligned}\mathcal{H}_{design} = &\sum_{i=1}^{N^{prot}} \sum_{j>(i+2)}^{N^{prot}} \alpha E_{sc}(r_{C\alpha_i C\alpha_j}) + \sum_{i=1}^{N^{prot}} \sum_{j=1}^{N^{b.s.}} \alpha E_{sc}(r_{C\alpha_i C\alpha_j}) + \\ &+ \sum_{i=1}^{N} \alpha E_{HOH} E_{sol}(\Omega - \Omega_i)\end{aligned} \tag{S6}$$

where the sum of the residue-residue interactions runs on intra-protein and inter-proteins only. Moreover, the minimum inter-protein distance has been chosen high enough to keep the proteins energetically decoupled, leaving protein Γ optimisation on the solvent interaction only. In this framework, Eq. S5 becomes a crucial ingredient since the constraint of maximising the number of permutations along the jointed sequence is the only coupling between the partners, therefore enforcing the competition during the design. Given the complex residue-residue interaction network of the compact structure of the protein, it is hard to find highly heterogeneous protein sequences with very low energy. Therefore, the design spontaneously isolate an optimal subset of amino acids for the protein design problem, thus leading as a consequence to extremely heterogeneous sequences for the protein Γ, so to maximise the overall permutation index.

We enhance the sampling by introducing an adaptive bias potential $W[E, \ln N_p, T]$ for each temperature, over two collective variables: E and N_p. $W[E, \ln N_p, T]$ is used to bias the acceptance probability of each Monte Carlo mutation move. Therefore, each Monte Carlo step is accepted with a probability

$$P_{acc}^{rep} = min\{1, \exp[\Delta W - (\Delta E - E_p \ln \frac{N_p^{new}}{N_p^{old}})/K_B T]\} \tag{S7}$$

or

$$P_{acc}^{swap} = min\{1, \exp[\Delta W - \Delta E/K_B T]\}, \tag{S8}$$

associated to the amino acid replacement and amino acids swap moves, respectively. In the latter equations ΔW, ΔE and $\ln \frac{N_p^{new}}{N_p^{old}}$ refer to the differences of the bias potential, the energy and the number of permutation between the new configuration and the old one; $E_p = 20\ K_B T$ is a scaling factor set to a value high enough to generate highly heterogeneous sequences (as in Ref. [2]).

The acceptance probability for the adaptive parallel tempering is:

$$P_{acc}^{Tswap} = min\{1, \exp[\Delta W + (\Delta E - E_p \ln \frac{N_p^{new}}{N_p^{old}})/K_B \Delta T]\}, \tag{S9}$$

where ΔW, ΔE and $\ln \frac{N_p^{new}}{N_p^{old}}$ refer to the differences of the relative quantities between the sequences belonging to replicas at different temperatures, and ΔT is the temperature difference.

Folding

We perform two different kind of folding simulations: the single-protein $\overline{G}$ folding (step IV in Fig.1) and the folding in presence of protein Γ (step V in Fig.1).

For the single-protein simulations, we apply the method fully described in Ref. [1, 2], that consist of Monte Carlo simulations with enhanced sampling techniques, namely the adaptive VMPT already introduced in the *Design* section.

The set of reduced temperatures for the parallel tempering (2.0; 1.8; 1.6; 1.4; 1.3; 1.2; 1.1; 1.0; 0.9; 0.8; 0.7; 0.65; 0.6; 0.55; 0.5; 0.45) is chosen in order to observe repeatedly folding events. The protein conformation is swapped periodically between replicas adjacent in temperature. We sample the protein conformations using crankshaft and pivot moves, to change the protein geometry.

As for the adaptive umbrella sampling, we construct a bias potential depending on two variables: the distance root mean square displacement $DRMSD$ and the number of hydrogen bonds HB. For HB we consider all the pairs of oxygen and hydrogen atoms that are closer than 2.5 Å, are at a distance along the chain larger than second neighbours and their respective residues are facing each other (i.e. $(\cos(\theta_1)\cos(\theta_2))^\nu$ in Eq. S2 differs from zero). The $DRMSD$ is a measure of the distance of a configuration from a target structure:

$$DRMSD = \sqrt{\frac{1}{C}\sum_{ij}(|\Delta\vec{r_{ij}}| - |\Delta\vec{r_{ij}}^{T}|)^2}, \tag{S10}$$

where the sum runs over the ij pairs in contact in the target structure, namely the native contacts C, identified using a cut off of 17 Å according to Ref. [1, 2]. $\Delta\vec{r_{ij}}$ is the distance between the residues i and j and $\Delta\vec{r_{ij}}^{T}$ is the same distance calculated over the target structure. As target, we use the crystallographic structure from the Protein Data Bank (PDB ID: 1pgb). We preferred the DRMSD measure instead of the classical RMSD, because it is more efficient in a Monte Carlo based code where few atoms are updated at each move. The values in Eq. S10 can be updated on the flight providing only the new distances.

For the simulations in presence of protein Γ, we adapted the procedure in order to handle a system composed of interacting partners. The simulation is performed in a cubic box (as shown in Fig. S6) containing two replicas of protein Γ (box side $\sim$ 360 Å), that are the mirror image of the other with respect to the xy plane passing through the centre of the box.

The proteins Γ are frozen in the box, and protein $\overline{G}$ starts the folding from a fully stretched conformation. An impenetrable slab region is defined between the proteins Γ to prevent the protein $\overline{G}$ to approach them from the convex side instead of the concave one generated with the moulding procedure.

Additionally to crankshaft and pivot, we perform rotation, translation and mirroring moves, to let the protein $\overline{G}$ reorient, diffuse in the box and switch from right to left handed conformations. The simulation is performed in parallel at 32 different temperatures and the replicas exchange information through the VMPT bias scheme [3]. The set of reduced temperatures (8.5; 7.8; 7.2; 6.6; 6.0; 5.4; 4.9; 4.6; 4.3; 3.9; 3.5; 3.1; 2.8; 2.55; 2.35; 2.2; 2.05; 1.9; 1.75; 1.6; 1.45; 1.3; 1.1; 1.0; 0.98; 0.95; 0.92; 0.88; 0.85; 0.82; 0.79; 0.76) is chosen so to observe the protein $\overline{G}$ repeatedly detaching from the protein Γ.

Each replica sampling is enhanced by a bias potential depending on two collective variables, namely the distance root mean square displacement within the protein $DRMSD_{intra}$, and between proteins, $DRMSD_{inter}$. Both the $DRMSD$s are evaluated according to Eq. S10, with the following specifications: accordingly, the $DRMSD_{intra}$ is computed adopting the native and isolated protein conformation as a target structure, while the $DRMSD_{inter}$ is computed using as a target structure the native protein bound to the protein Γ; the normalisation factor C is the number of native contacts for $DRMSD_{inter}$ and twice the number of native contacts for $DRMSD_{intra}$; $\Delta \vec{r_{ij}}$ for $DRMSD_{inter}$ is evaluated with respect to the protein Γ closest to the protein $\overline{G}$ for each ij pair.

Association constant

For the evaluation of the association constant $K_a = \frac{Q_b}{Q_f}\frac{V_{box}}{n}$, we need to assess the number of protein Γ in the box $n = 2$, define the accessible simulation volume V_{box} and discern bulk solution configurations (contributing to Q_f, the partition function of the free protein) from the ones where the protein $\overline{G}$ is directly in contact with the protein Γ (contributing to Q_b, the partition function of the bound configurations). The accessible volume is easily obtained by subtracting the hard wall contribution to the box volume. The bound configurations are the ones characterised by a protein-protein interaction energy $E_{inter} \neq 0$. The configurations contributing to Q_f, instead, are the ones for which the central amino acid of the protein is at a distance $r > H_{lenght} + R_{int}$ with respect to all the amino acids of the surface of

protein Γ (being H_{lenght} half of the stretched protein length; R_{int} the interaction radius) and, therefore, where the interaction with protein Γ is not possible. We refer to the latter region as the bulk solution.

SOLUTION BASINS

For all scenarios, we generated a large number of sequences that we group in solution basins. In Fig. S4 we plot the distribution of the Hamming distances calculated for all the possible pairs between the basins. The latter is measured using the Hamming distance that determines the difference between two sequences of equal length by counting the number of substitutions needed to make them coincide. It is often more convenient to normalise the Hamming distance by dividing by the protein length N. For all scenario we observe large number of solutions that differ for more than 50% of the residues.

A crucial feature of the distributions in Fig. S4, are the peaks at high Hamming distances. They demonstrate that the protein-protein coupling induces the design process to explore distinct solution basins. Given that the proteins are energetically decoupled (evident from the low value of E_{sc}-inter that ranges from -1.4 to -2.7 k_BT), the influence of the protein Γ can be attributed to the maximisation of the letter permutations expressed by Eq. (S5). Such coupling enforces an increasing competition between the proteins for the alphabet available while increasing the size of protein Γ.

However, ζ= 0.60 and 0.80 have also a significant number of related solutions with sequences differing for 20% only (see Fig. S4(f)). To check that nevertheless the ζ= 0.60 and ζ= 0.80 basins are distinct, we also calculated the self-overlap distributions for ζ= 0.60 and ζ= 0.80 (see Fig. S3) and we obtained profiles that are different from each other. More importantly, they differ from the one plotted in Fig. S4(f), demonstrating that although there are similarities, the two basins are distinct.

It is important to notice that the significant difference between the sequences is an evidence of the substantial effect of the coupling established by the maximisation of the total permutations N_P. The distance between the ζ 0.60 and ζ 0.80 solutions is considerably smaller than between the others. However, we verified that they correspond to distinct solution basins.

We select one sequence from each basin, that we consider representative of the whole

basin. The selected sequences are shown in Tab. S1. In Fig. S11 we plot the occurrence frequency of each amino acid type in the selected sequences.

Given that the caterpillar alphabet is composed by 18 letters, the frequency of each amino acid in a maximally heterogeneous sequence would be $1/18 \sim 6\%$. Therefore, we set a threshold at a slightly higher value=10%, to safely discern the contribution of dominating amino acids from the random occurrences. From the plot, it becomes apparent that the protein $\overline{G}$ designed sequence has a restricted composition of amino acids leading to a reduced effective protein alphabet.

ζ **0.20** SGGGGGGRKKKKKYYYVVVVVVGSSGGVKKKKKGGNYYYYYYYYVYVVKKKKGGGR

ζ **0.40** FGGGGGGKKRRKVYYVKKKRRRGFFYYYYKKRRWRNFYYYYVVKKKKVYYGGGGGG

ζ **0.60** FFFFGDGGYYYYYKKKKHHHHIRRRRRRFYYYYGGGRKKKKKKKYHHHRFFFGGGG

ζ **0.80** FFFFFGGGYYYYVKKKHHHHHKIIRRRRRFYYYGGGTKKLKKKYHHHRRRFFGGGG

TABLE S1. protein $\overline{G}$ designed sequences for the investigated systems.

ζ	**0.20**	**0.40**	**0.60**	**0.80**
**0.20**	0	61	87	86
**0.40**		0	84	86
**0.60**			0	27
**0.80**				0

TABLE S2. Hamming distance [%] between designed protein sequences in Tab. S1.

* icoluzza@cicbiomagune.es

[1] I. Coluzza, PLoS ONE **9**, e112852 (2014).

[2] I. Coluzza, PLoS ONE **6**, e20853 (2011).

[3] I. Coluzza and D. Frenkel, ChemPhysChem **6**, 1779 (2005).

[4] A. Irbäck, F. Sjunnesson, and S. Wallin, Proceedings of the National Academy of Sciences of the United States of America **97**, 13614 (2000).

[5] J. Garnier and B. Robson, *Prediction of Protein Structure and the Principles of Protein Conformation*, edited by G. D. Fasman (Springer US, Boston, MA, 1989) p. 798.

[6] V. Bianco, G. Franzese, C. Dellago, and I. Coluzza, Physical Review X **7**, 21047 (2017).

[7] C. Cardelli, V. Bianco, L. Rovigatti, F. Nerattini, L. Tubiana, C. Dellago, and I. Coluzza, Scientific Reports **7**, 4986 (2017).

[8] I. Coluzza and D. Frenkel, Biophysical journal **92**, 1150 (2007).

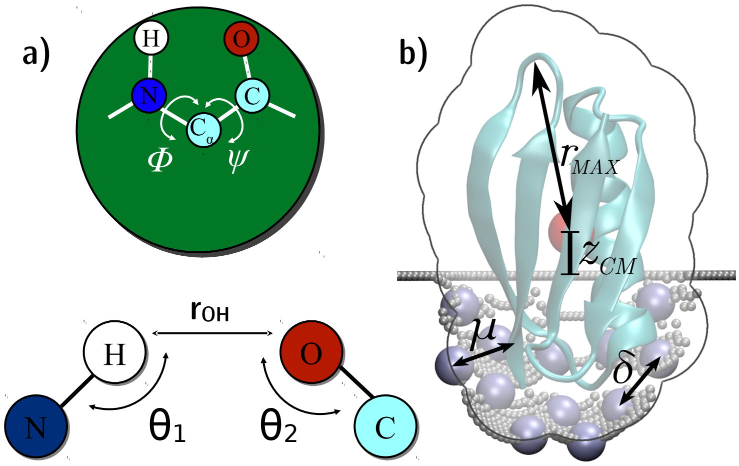

S1. **a)** Caterpillar protein model: the green circle represents the amino acid self-avoidance volume, which has a radius of 2 Å and is centred on the position of the C_α atom. Each amino acid is represented through backbone atoms only. Side chain-side chain interactions are represented via a square-well-like potential (Eq. S3).For the hydrogen bonds, the model include a $10-12$ Lennard-Jones potential, that is a function of the distance r_{OH}, and tunes it with a multiplicative factor that involves the angles θ_1 and θ_2, so to account for the directionality of the bond. **b)** Artificial protein Γ parameters. Gray dots are self-avoiding beads; blue spheres are $C_{\texttt{Surf}}$ atoms, i.e. C_α atoms of the protein Γ. The red spot represents the centre of mass of the protein, z_{CM} is its height with respect to the $z=0$ plane and r_{MAX} is the maximum CM-C_α distance. μ is the minimum C_α protein-C_α surface distance, i.e. the value one has to use to inflate the protein for shaping protein Γ. δ is the minimum distance between two activated C_α of protein Γ.

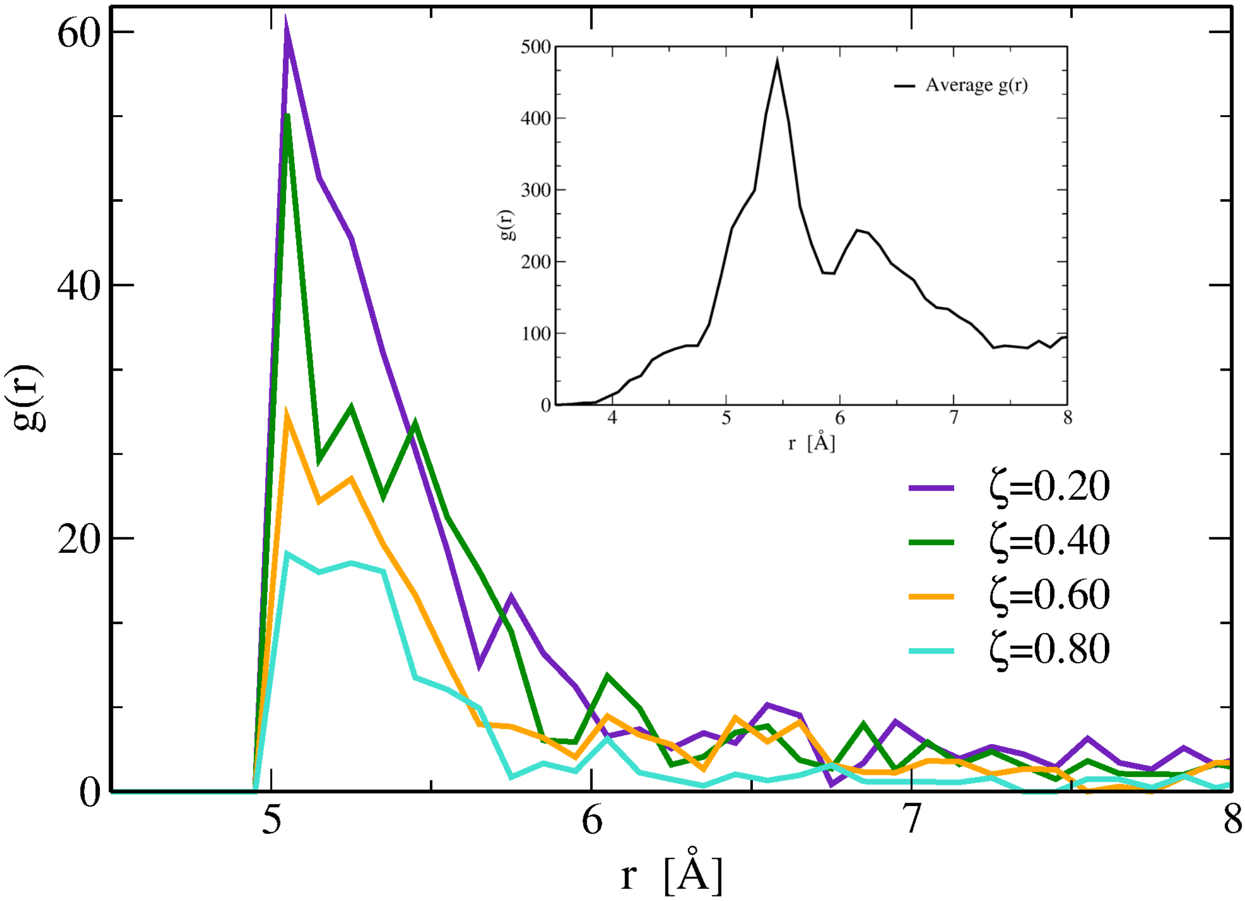

S2. Radial distribution function $g(r)$ calculated over the active residues $C_{\texttt{Surf}}$ of the protein Γ placed at the minimum distance $\delta = 5$ Å from each other. The latter was determined by identifying the value of δ that yields the mean nearest distance $\int_0^{7.5} \rho 4\pi r^2 g(r) r \, dr$ closest to 5.7 Å obtained by averaging over 145 protein structures taken from the PDB (black line in the inset). The integral interval was chosen visually taking a region large enough to fully contain the first peak in the $g(r)$. It is important to stress that in the protein $g(r)$ the selected distribution includes the contribution from all secondary structure elements [7].

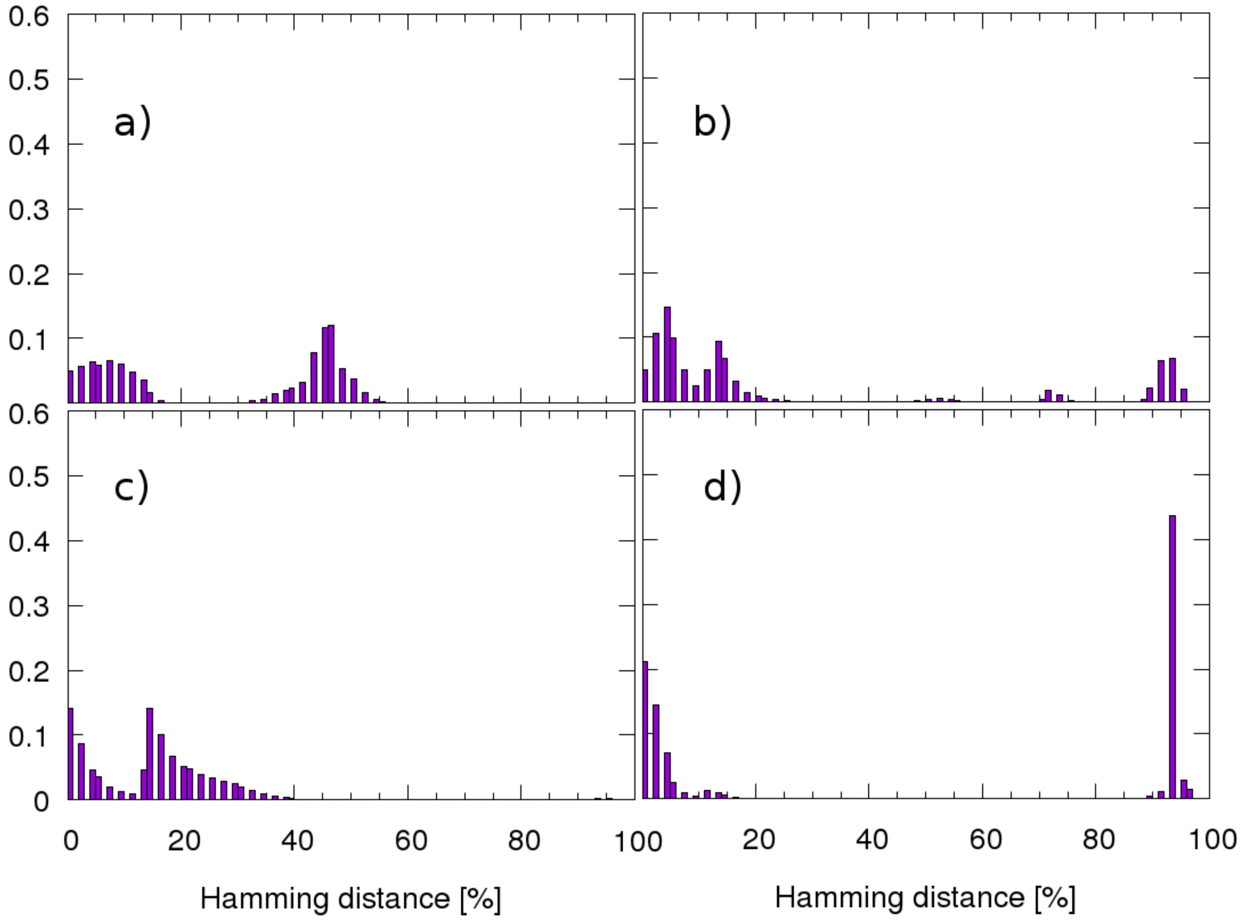

S3. Histograms obtained by evaluating the Hamming distance (% relative to the chain length) between all possible pairs of sequences chosen selecting 200000 solutions around the design free energy minimum of systems A and B, corresponding to the ζ values specified in the following. All panels refer to self comparison of sequences belonging to the same basin. a) A: $\zeta = 0.20$; B: $\zeta = 0.20$ b) A: $\zeta = 0.40$; B: $\zeta = 0.40$ c) A: $\zeta = 0.60$; B: $\zeta = 0.60$ d) A: $\zeta = 0.80$; B: $\zeta = 0.80$

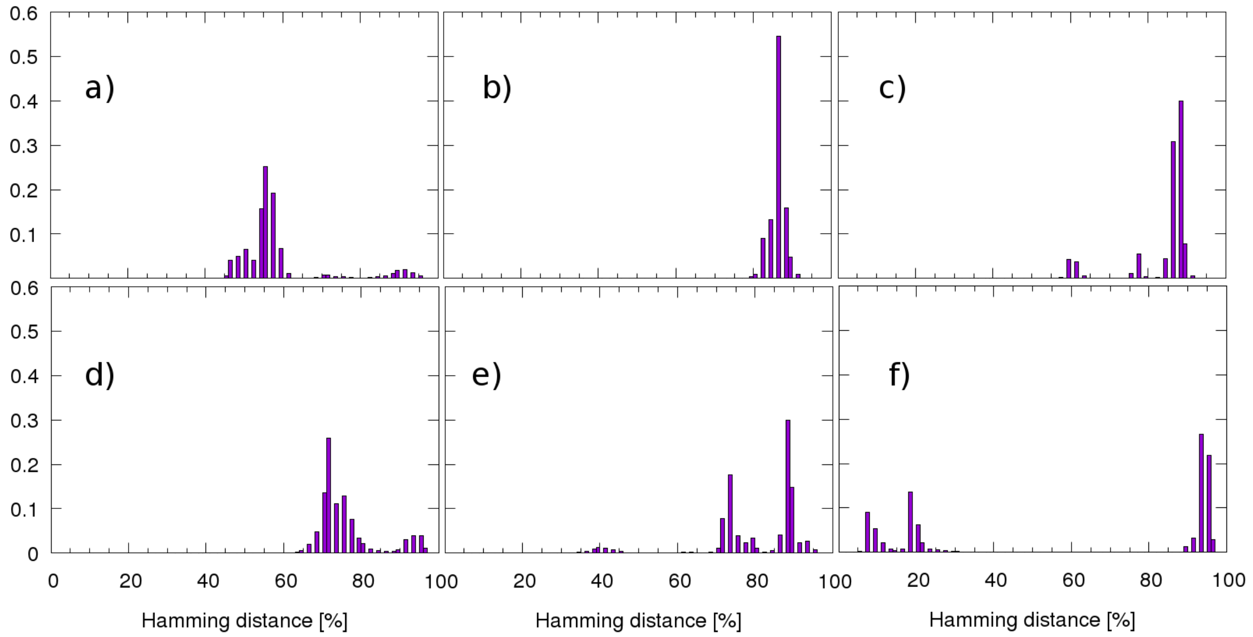

S4. Histograms obtained by evaluating the Hamming distance (% relative to the chain length) between all possible pairs of sequences chosen selecting 200000 solutions around the design free energy minimum of systems A and B, corresponding to the ζ values specified in the following. a) A: ζ= 0.20; B: ζ= 0.40 b) A: ζ= 0.20; B: ζ= 0.60 c) A: ζ= 0.20; B: ζ= 0.80 d) A: ζ= 0.40; B: ζ= 0.60 e) A: ζ= 0.40; B: ζ=0.80 f) A: ζ= 0.60; B: ζ= 0.80

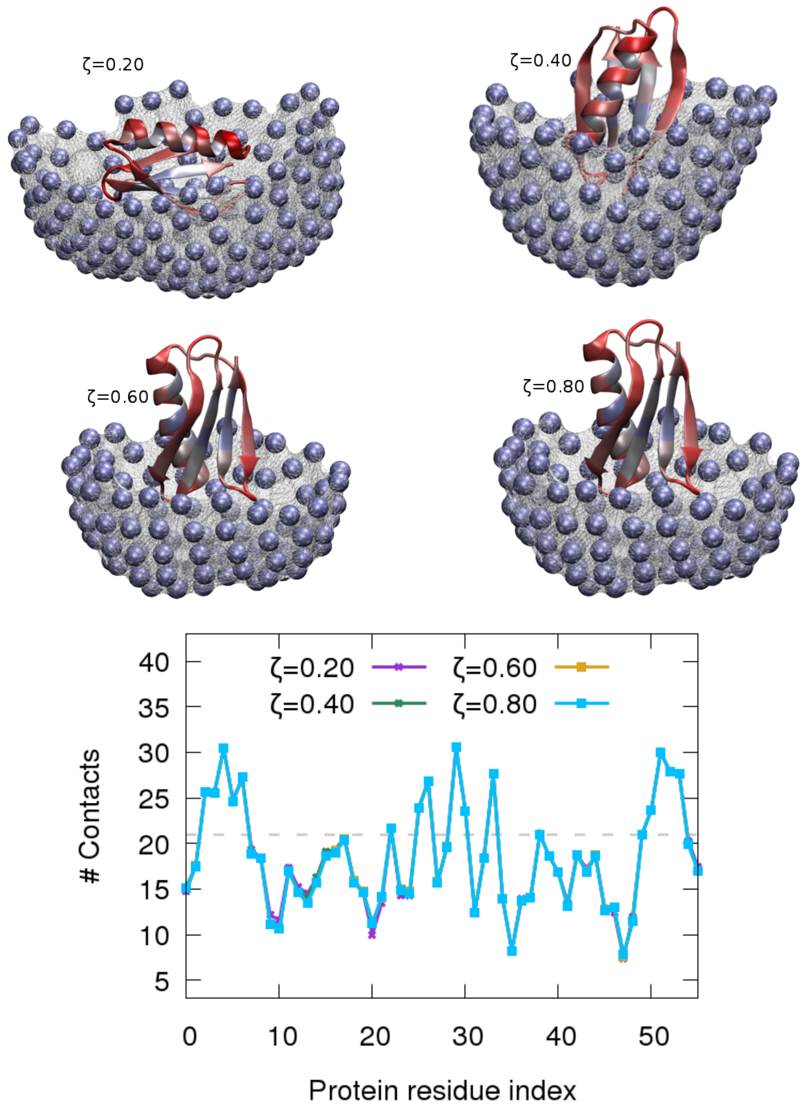

S5. Water exposure profiles of protein G in presence of proteins Γ constructed with different ζ values. The colour scheme of the protein is related to the number of total contacts: solvent exposed regions in red, buried in blue. The plot shows the number of contacts for each protein residue. The grey dashed line defines the threshold $\Omega = 21$ on the number of neighbours above which an amino acid is considered buried in the protein core. We used the information relative to the exposure of protein G to set the radius of the probe sphere used in the evaluation of the number of contacts for each active C_α of the protein Γ (the blue spheres in the figure). We counted the offset for the number of neighbours for each of the protein Γ C_α by multiplying the fraction of the volume of a sphere of radius 6 Å that lies under the mesh surface by the average amino acid density in globular proteins ($\rho = 0.011687\ aa/\text{Å}^3$, evaluated over a set of 145 globular proteins). The radius of the sphere was optimized to reach an average exposure of each residue similar to the one of the most exposed residues of protein G (corresponding to the minima in the plot).

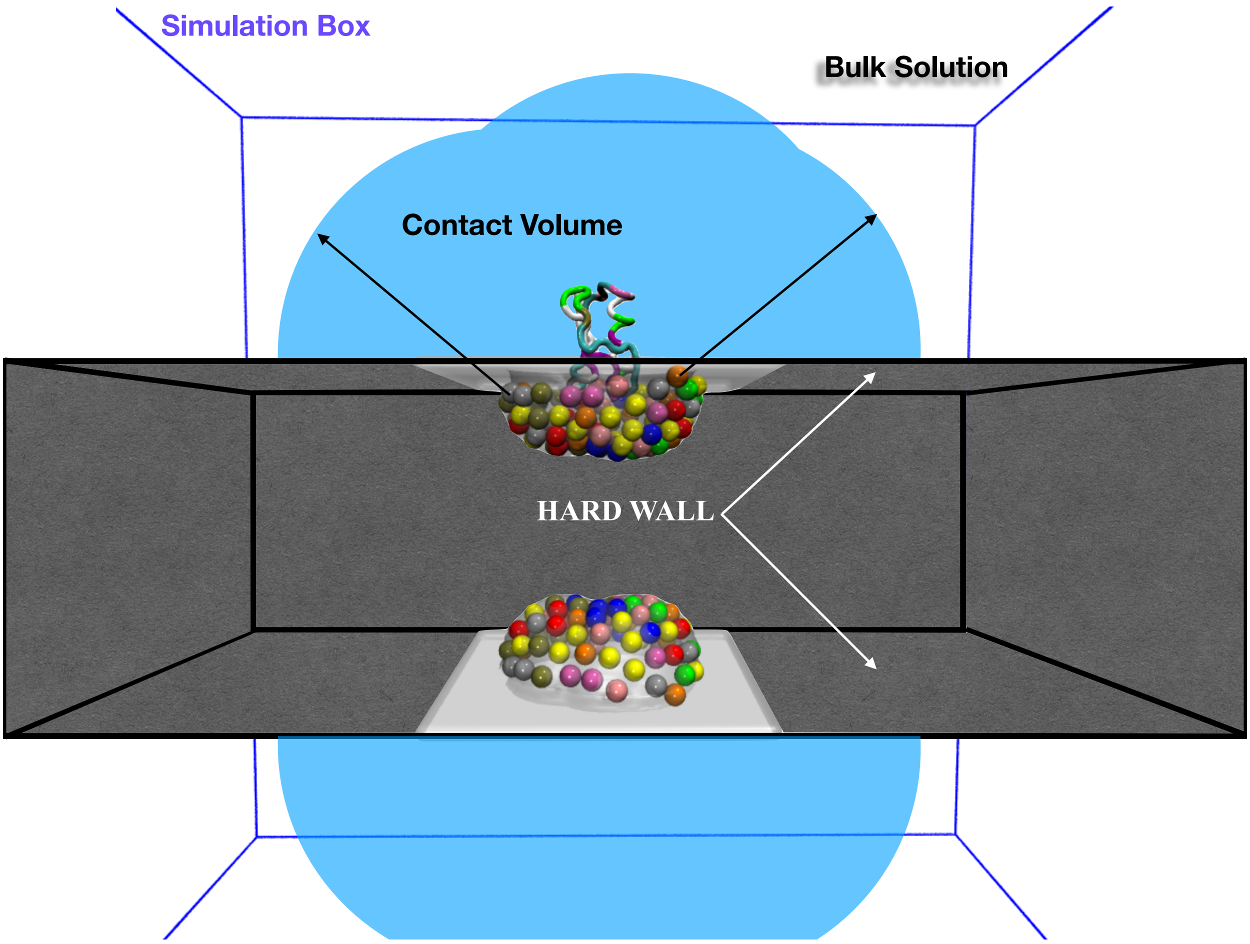

S6. Simulation box for protein folding in presence of two copies of the protein Γ. The protein Γ is a surface decorated with $C\alpha$ atoms, i.e. the coloured spheres in the image. Each colour, both on the protein backbone and on the $C\alpha$ of the surface of protein Γ, represents a different amino acid type. The proteins sequence are designed simultaneously with the procedure described in the *Design* sub-section. The protein is a flexible polymer diffusing in the box under Periodic Boundary Conditions. A mirroring move provides a swap between opposite chiralities, since the caterpillar model does not take into account the amino acid chirality. The cubic box contains two proteins Γ, one the mirror image of the other, the position of which is fixed during the simulation. The proteins Γ are far apart enough to prevent the protein G to interact with both at the same time. A hard wall between the protein Γ prevents the protein G to approach the surfaces from the convex side.

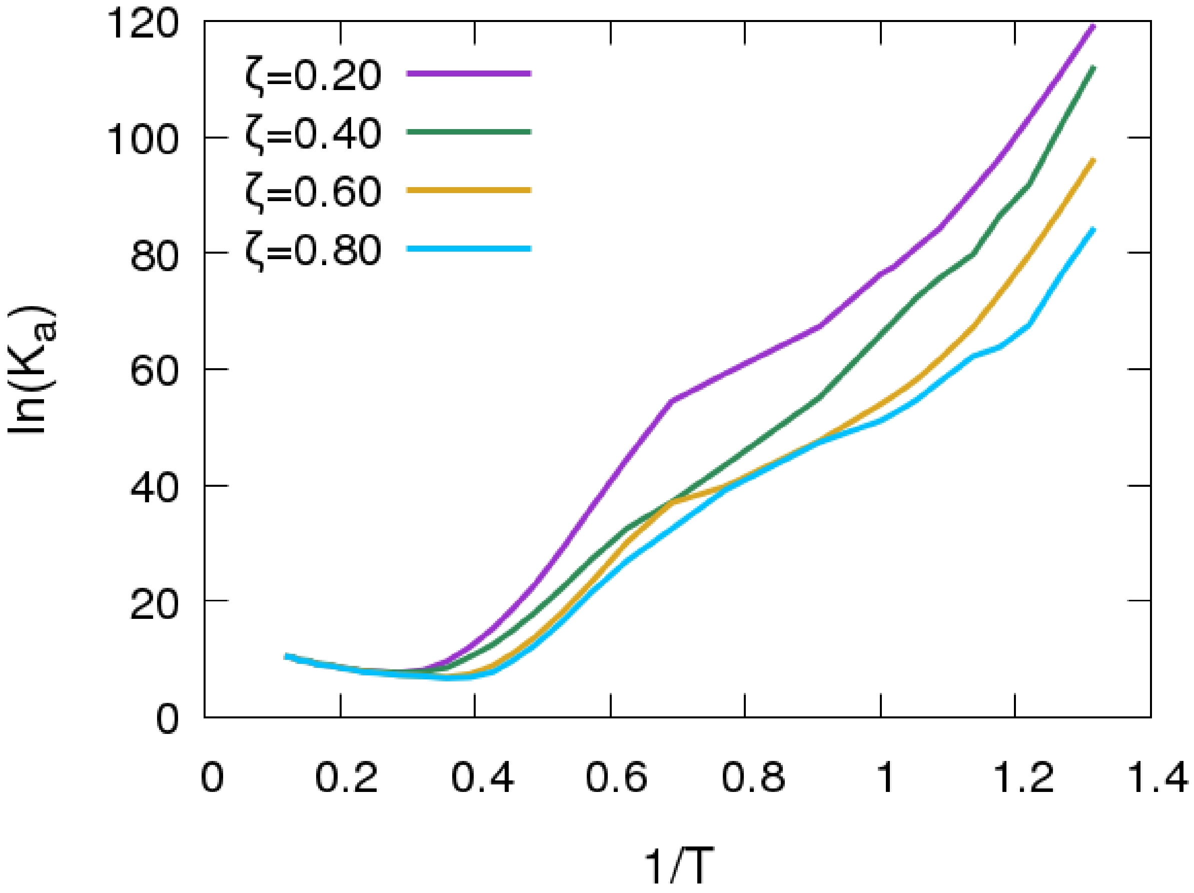

S7. Van't Hoff plot of $\ln K_a$ as a function of the inverse reduced temperature $1/T$ for the investigated systems. The association constant $[l/mol]$ is computed as $K_a = \exp(-\Delta F/k_B T) V_{box}/n$, where V_{box} is the accessible volume of our simulation box, $n = 2$ is the number of proteins Γ and $\Delta F = -k_B T \ln(Q_b/Q_f)$ is the binding free energy [8]. The partition functions Q_b and Q_f refer to all protein conformations bound to protein Γ and free in the bulk solution, respectively.

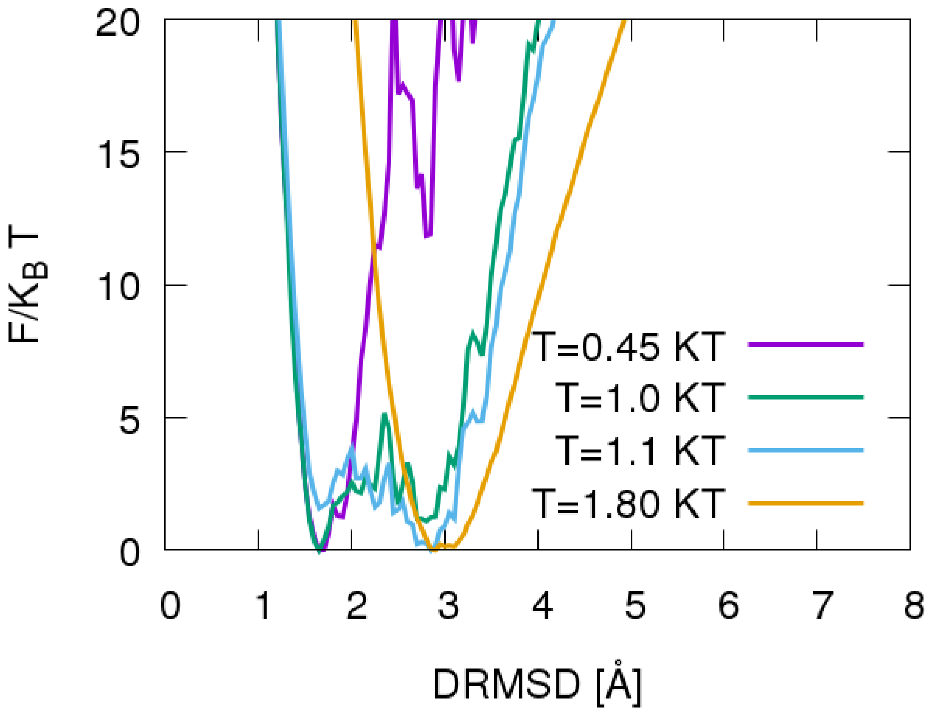

S8. Folding free energy profiles F/k_BT at different temperatures for simulations of protein $\overline{G}$ designed for the binding site at $\zeta = 0.80$ placed in an empty box. The folding temperature is between 1.0 and 1.1, since is defined as the temperature at which there is equilibrium between the folding in the native structure (configurations in the minimum below 2 Åin DRMSD) and a random globule (configurations corresponding to the minimum above 2 Å).

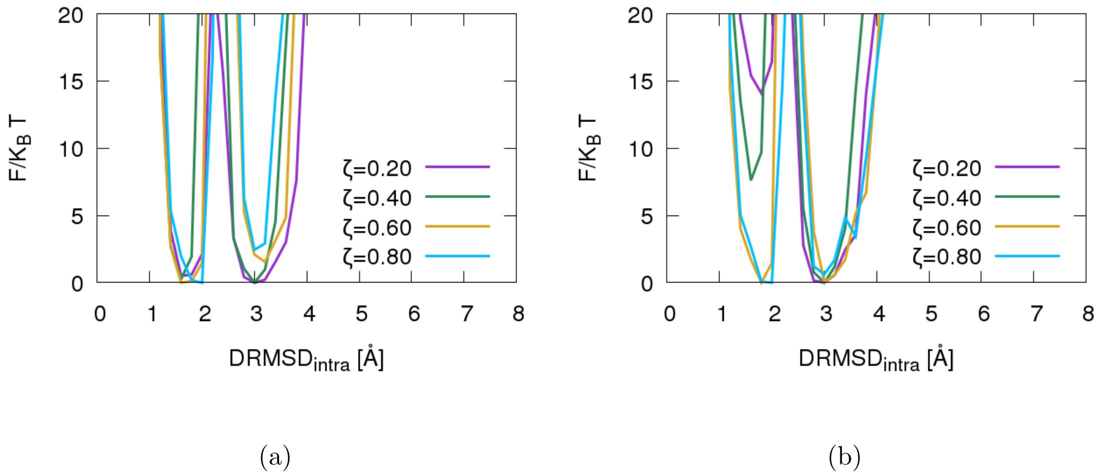

S9. Folding free energy profiles F/k_BT at reduced temperature 0.76 (panel a) and 1.00 (panel b) as a function of DRMSD intra protein $\overline{G}$ from the native protein G structure. Simulations of protein $\overline{G}$ in presence of protein Γ for $\zeta = (0.20, 0.40, 0.60, 0.80)$. The curves have been evaluated on bound configurations, i.e. where protein $\overline{G}$ is directly interacting with protein Γ. Panel a): The presence of the two equivalent minima suggests that the protein binds when properly folded as well as in misfolded states with the same probability. Panel b): upon increasing the temperature, systems characterised by larger protein Γ surfaces undergo to a destabilisation of the properly folded states.

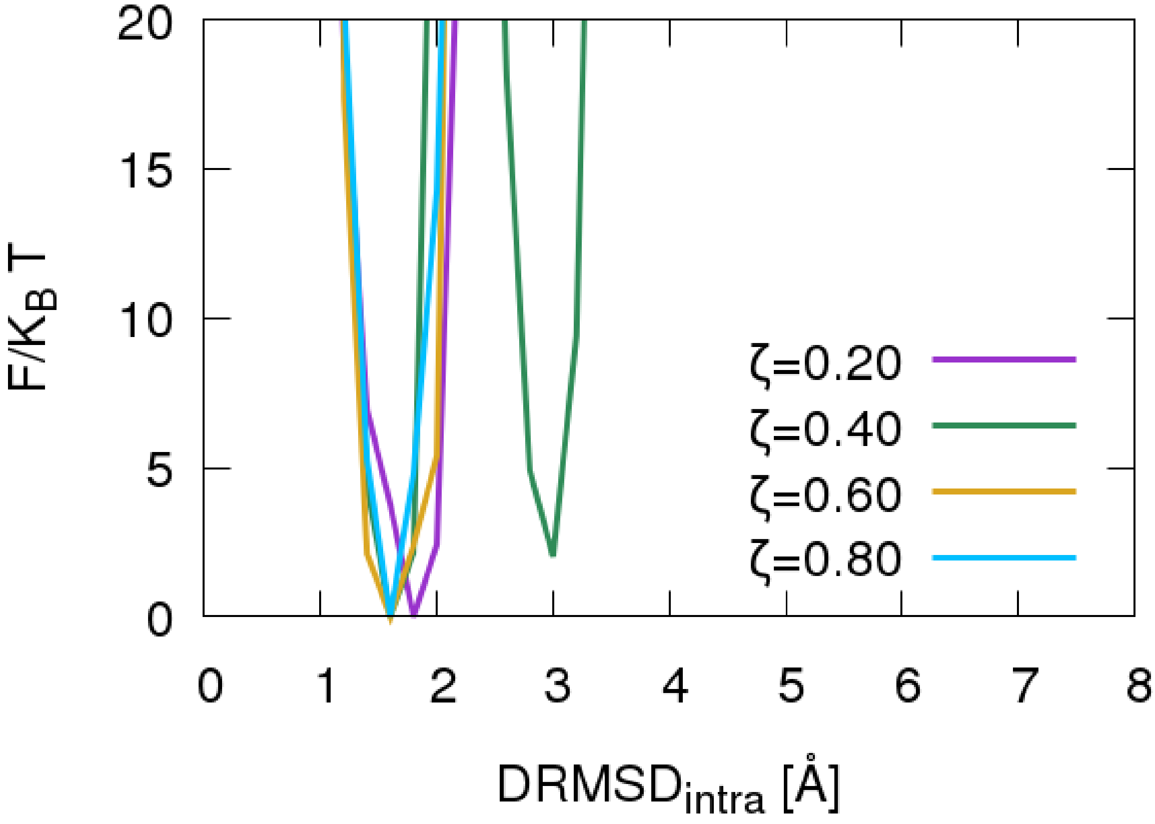

S10. Folding free energy profiles F/k_BT at reduced temperature 0.76 as a function of $DRMSD_{intra}$ protein $\overline{G}$ from the native target structure. Simulations of protein $\overline{G}$ in presence of protein Γ for $\zeta = (0.20, 0.40, 0.60, 0.80)$. The curves have been evaluated on configurations in the bulk solution, where the protein-protein distance is larger than the interaction one. The presence of a minimum below $DRMSD$ =2 Å shows that the protein is folded in the bulk.

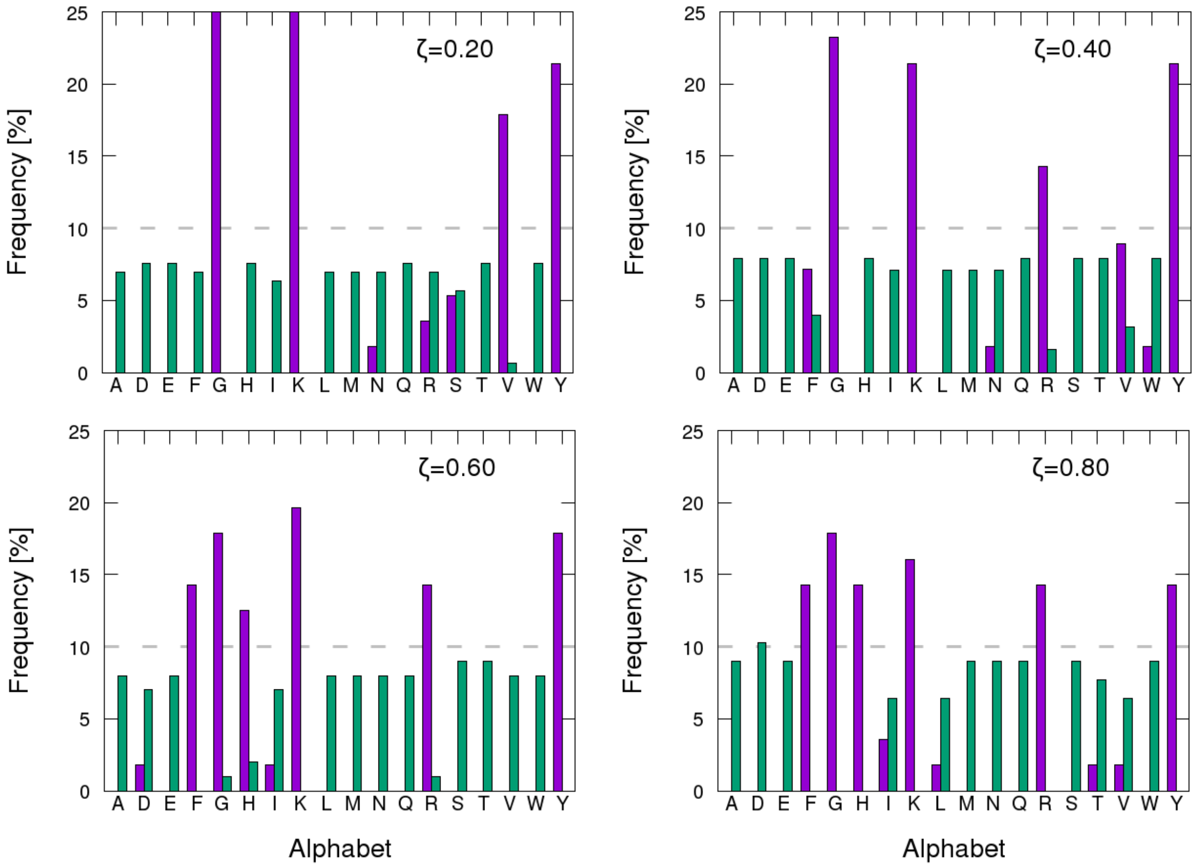

S11. Frequency of amino acids in the designed sequences. The grey dotted line is the value used to consider one amino acid in the effective alphabet. Purple bars refer to the sequence of protein $\overline{G}$, while green bars to the sequences of protein Γ. Cysteine (C) and Proline (P) are not included in the design alphabet.
